# LncRNA TUSC7 Inhibits Cell Proliferation in Chronic Lymphocytic Leukemia by Modulating the miR‐211‐5p/SLC37A3 Axis

**DOI:** 10.1002/kjm2.70003

**Published:** 2025-03-08

**Authors:** Xu‐Li Wang, Jia Mei

**Affiliations:** ^1^ Department of Hematology, Jinling Hospital, Affiliated Hospital of Medical School Nanjing University Nanjing China; ^2^ Department of Pathology, the Second Hospital of Nanjing Affiliated to Nanjing University of Chinese Medicine Nanjing China

**Keywords:** ceRNA, chronic lymphocytic leukemia, miR‐211‐5p, SLC37A3, TUSC7

## Abstract

Chronic lymphocytic leukemia (CLL) is a malignant lymphoproliferative disorder. Long non‐coding RNAs (lncRNAs) have been implicated in various regulatory processes and cancer development. Among these, lncRNA tumor suppressor candidate 7 (TUSC7) has been identified as a tumor suppressor gene. We herein measured TUSC7 expression using RT‐qPCR and investigated its biological role in CLL through gain‐of‐function experiments. Our results revealed that TUSC7 expression was significantly lower in CLL patients compared to healthy controls, and its downregulation was associated with poor prognosis. Meanwhile, TUSC7 overexpression inhibited cell proliferation while promoting cell apoptosis. Mechanistically, TUSC7 interacted with miR‐211‐5p, thereby regulating the downstream target gene, solute carrier family 37 member 3 (SLC37A3). Further rescue experiments demonstrated that silencing SLC37A3 or upregulating miR‐211‐5p reversed the effects of TUSC7 elevation on cell proliferation and apoptosis. In conclusion, our findings suggest that TUSC7 regulates cell proliferation in CLL through the miR‐211‐5p/SLC37A3 axis.

## 1 Introduction

Chronic lymphocytic leukemia (CLL), the most prevalent form of leukemia, accounts for approximately 30% of all leukemia cases in adults, with an incidence of 3–5 per 100,000 in developed countries [1]. The disease primarily affects the elderly, with the median age at diagnosis around 70 years. A slight male predominance has also been observed [2]. The pathogenesis of CLL is influenced by a combination of genetic factors, medical history, and occupational exposures [3, 4, 5]. In the early stages, many individuals with CLL are asymptomatic. As the disease progresses, however, patients may experience symptoms such as painless lymphadenopathy, fatigue, fever, upper left abdominal discomfort (often due to splenomegaly), night sweats, weight loss, and recurrent infections [6]. Although the precise etiology of CLL remains elusive, several risk factors have been identified, including age, race, a family history of hematologic malignancies, exposure to certain chemicals, and conditions leading to lymphocyte proliferation [7]. The clonal expansion of morphologically mature B‐lymphocytes, which accumulate in secondary lymphoid tissues, blood, and bone marrow, leads to splenomegaly, lymphocytosis, bone marrow infiltration, and lymphadenopathy. This process is a hallmark of CLL pathogenesis [8]. Clinically, CLL is primarily categorized into two forms: the aggressive form, characterized by mutations in the immunoglobulin heavy chain variable region (IGHV), and the indolent form, associated with unmutated IGHV [9, 10]. Currently, the combination of fludarabine, cyclophosphamide, and rituximab (FCR) chemoimmunotherapy is considered the gold standard frontline treatment for CLL [11]. However, the rate of relapse remains high. While the majority of CLL patients experience an indolent clinical course, often requiring no or delayed therapy and demonstrating prolonged survival, others exhibit aggressive disease that necessitates early diagnosis and intervention to prevent frequent relapses [12, 13]. Consequently, identifying reliable biomarkers for CLL and elucidating their regulatory mechanisms is of great significance.

Noncoding RNAs (ncRNAs) play pivotal roles in the pathogenesis of various cancers. Among them, long noncoding RNAs (lncRNAs), defined as ncRNAs longer than 200 nucleotides, have emerged as critical regulators of cellular processes in cancer [14, 15]. Recent studies have highlighted the involvement of lncRNAs in CLL progression. For instance, LEF1‐AS1 promotes CLL cell proliferation and inhibits apoptosis by upregulating the LEF1 level [16], while lncRNA CRNDE suppresses cell proliferation via the miR‐28/NDRG2 axis in CLL [17]. These findings underscore the importance of elucidating the precise mechanisms by which lncRNAs regulate cell proliferation and apoptosis in CLL. One such candidate, tumor suppressor candidate 7 (TUSC7), is a lncRNA with four exons exceeding 2 kb in length, located at chromosome 3q13.31. TUSC7 has been characterized as a novel tumor suppressor in multiple cancers, including osteosarcoma [18, 19], endometrial carcinoma [20], colorectal cancer [21, 22], and pancreatic carcinoma [23]. However, the biological functions of TUSC7 and its underlying mechanisms in CLL progression remain poorly understood.

MicroRNAs (miRNAs) are a class of small non‐coding RNAs that regulate mRNA expression by binding to the 3′untranslated regio (3′UTR) of target mRNAs. Several studies have demonstrated that miRNAs play pivotal roles in cancer biology, including the regulation of cell proliferation, apoptosis, differentiation, and invasion [24, 25]. Accumulating evidence suggests that lncRNAs interact with miRNAs, acting either as competitive endogenous RNAs (ceRNAs) or as miRNA sponges [26]. Based on this, the present study proposes a regulatory mechanism involving the lncRNA‐miRNA‐mRNA axis in CLL.

The aim of this study is to investigate the role and underlying regulatory mechanism of TUSC7 in CLL. We hypothesize that TUSC7 may function as a potential tumor suppressor in CLL. Our findings could support the exploration of TUSC7 as a novel therapeutic target for CLL.

## 2 Materials and Results

### 2.1 Patient Samples

Peripheral blood samples were collected from 40 untreated CLL patients and 40 healthy volunteers, with informed consent from the participants and approval from the Ethics Committee of The Second Hospital of Nanjing, Affiliated to Nanjing University of Chinese Medicine. Peripheral blood mononuclear cells (PBMCs) were isolated from the blood samples using density gradient centrifugation with Lymphoprep (Stemcell technologies, Canada). The clinical significance of lncRNA TUSC7 expression was evaluated in relation to demographic characteristics and prognostic factors, including gender, age at diagnosis, Binet stage, CD38 status, cytogenetic risk, somatic hypermutational status of immunoglobulin heavy variable genes (IGHV SHM) status, TP53 mutational status, the International Prognostic Index for CLL (CLL‐IPI), B symptoms, treatment history, and laboratory parameters (white blood cell count [WBC], lymphocyte count [Ly], platelet count, hemoglobin [Hb], β2‐microglobulin [β2M], and lactate dehydrogenase [LDH]), as summarized in Table 1.

**TABLE 1:** Clinicopathological characteristics of CLL patients in relation to lncRNA TUSC7 expression.

Variables	Total (*n* = 40)	TUSC7^low^ group (*n* = 20)	TUSC7^high^ group (*n* = 20)	*p*
Gender, *n*				
Male	28	17	11	0.038
Female	12	3	9	
Age of onset, *n*				
< 65	26	12	14	0.507
≥ 65	14	8	6	
Binet stage, *n*				
A	18	6	12	0.086
B	16	9	7	
C	6	5	1	
CD38 status, *n*				
Positive (≥ 30%)	12	10	4	0.047
Negative (< 30%)	28	10	16	
Cytogenetic risk, *n*				
Favorable (del13q14)	13	6	7	0.076
Intermediate (no aberrations, trisomy 12)	17	6	11	
Unfavorable (del11q22–23, del17p13)	10	8	2	
IGHV SHM status, *n*				
Mutated	21	11	10	0.752
Unmutated	19	9	10	
TP53 mutational status, *n*				
wt	32	14	18	0.114
Mutated	8	6	2	
CLL‐IPI, *n*				
Low (score 0–1)	18	5	13	0.034
Intermediate (score 2–3)	11	6	5	
High (score 4–6)	8	6	2	
Very high (score 7–10)	3	3	0	
B symptoms				
No	25	9	16	0.022
Yes	15	11	4	
Treatment requirements				
No	8	2	6	0.114
Yes	32	18	14	
WBC [×10^9^/L], median (range)	38.4 (7.3–536)	45.6 (10.5–536)	35.2 (7.3–485)	0.246
Ly [×10^9^/L], median (range)	29.3 (3.4–567.1)	34.8 (6.2–567.1)	27.5 (3.4–457.3)	0.221
Platelets [×10^9^/L], median (range)	171 (1–435)	143 (3–372)	196 (1–435)	0.048
Hb [g/L], median (range)	128 (44.3–184)	123.2 (44.3–164)	145.8 (83.2–184)	0.017
β2M [mg/L], median (range)	3.4 (0.6–10.2)	3.5 (0.6–10.2)	3.3 (2–7.3)	0.742
LDH level [U/L], median (range)	436.4 (154.3–1492.3)	513.3 (267.3–1492.3)	325.9 (154.3–863.7)	0.005

Abbreviations: β2M, β2‐microglobulin; CLL‐IPI, The International Prognostic Index for CLL; Hb, hemoglobin; LDH, lactate dehydrogenase; Ly, lymphocytes; IGHV SHM, somatic hypermutational status of immunoglobulin heavy variable genes; WBC, white blood cells; wt, wild type.

### 2.2 LncRNA‐miRNA Interaction

DIANA‐LncBase v2 (http://dianalab.e‐ce.uth.gr/) is a comprehensive databse designed to index miRNA targets on noncoding transcripts. The “Prediction module” offered valuable insights into the downstream miRNAs of TUSC7. The top 15 miRNAs predicted by DIANA are presented in Figure 2B. Additionally, the binding sites between TUSC7 and miR‐211‐5p are also depicted in the DIANA database.

### 2.3 miRNA‐mRNA Interaction

The miRDB database (https://mirdb.org/) is a comprehensive resource for miRNA target prediction. We utilized the miRDB database to predict potential miRNA‐mRNA interactions, identifying mRNAs with a target score of 100 as putative downstream targets of miR‐211‐5p. Additionally, the binding sites between miR‐211‐5p and SLC37A3 were predicted using the TargetScan tool (https://www.targetscan.org/).

### 2.4 Cell Treatment

CLL cell lines (JVM‐14, MEC‐1, LIZ1, KAT1) and peripheral blood lymphocytes (PBLs) (ATCC, Gaithersburg, MD, USA) were cultured in RPMI 1640 medium (90%, Sunncell, Hubei, China) supplemented with 10% FBS (Gibco, Waltham, MA, USA), 3% Phytohemagglutinin (PHA) (Maokangbio, Shanghai, China), 2% heparin (10 U/mL), 100 U/mL penicillin, and 100 mg/mL streptomycin at 37°C.

Short hairpin RNAs targeting SLC37A3 (sh‐SLC37A3) and its negative control (sh‐NC) were purchased from Genechem (Shanghai, China). MiR‐211‐5p mimic and NC mimic were synthesized by GenePharma (Shanghai, China). The full‐length sequences of TUSC7 were subcloned into the pcDNA3.1 vector (Sangon, Shanghai, China) to generate the pcDNA3.1/TUSC7 construct. JVM‐14 and MEC‐1 cells were transfected with 1 μg of TUSC7 overexpression vectors, or 50 nM miR‐211‐5p mimics, or 50 nM sh‐SLC37A3, or their corresponding controls using Lipofectamine 2000 (Invitrogen, Carlsbad, CA, USA). After 48 h of transfection, the cells were collected for subsequent analysis.

### 2.5 RT‐qPCR

Total RNA was extracted from PBMCs or CLL cell lines using Trizol reagent (Invitrogen) and subsequently reverse transcribed into cDNAs using the PrimeScript RT Reagent Kit (Takara, Liaoning, China). RT‐qPCR was performed with the SYBR Premix Ex TaqTM II reagent kit (RR820A, Takara) on a Light Cycler 480 instrument (Roche, Basel, Switzerland). GAPDH and U6 were used as internal references. Relative quantification was determined using the 2^−ΔΔCT^. Premier sequences are provided in Table 2.

**TABLE 2:** Sequences of primers used for reverse transcription‐quantitative PCR.

Gene	Sequence (5′➔3′)
TUSC7 forward	CATGGGAAACAGAAGGCAC
TUSC7 reverse	CTCAGAGGTCAAACAGGCA
miR‐211‐5p forward	ACACTCCAGCTGGGCAAGTAGCATCAACTA
miR‐211‐5p reverse	TGGTGTCGTGGAGTCG
miR‐204‐5p forward	GCCAGATCTGGAAGAAGATGGTGGTTAGT
miR‐204‐5p reverse	GGCGAATTCACAGTTGCCTACAGTATTCA
SLC37A3 forward	AGCAGCAACCATTTGTTCC
SLC37A3 reverse	GATGAATAGGCCCACAGCA
TCF12 forward	AGCTTCAATGGTTGGAACTC
TCF12 reverse	GTAGTGACTGTACTAGACAGGA
RAB22A forward	AAACATCAACCCAACAATAGGG
RAB22A reverse	GTGCTAAGGCACGAAATCG
ANKRD13A forward	AATGTTAATGGCTGTAGCACTG
ANKRD13A reverse	GATGTGGGAAGCTGAATCAG
U6 forward	TGCGGGTGCTCGCTTCGGCAGC
U6 reverse	CCAGTGCAGGGTCCGAGGT
GAPDH forward	TCAAGATCATCAGCAATGCC
GAPDH reverse	CGATACCAAAGTTGTCATGGA

### 2.6 CCK‐8

Cells were seeded into 96‐well plates (1 × 10^3^ cells/well). At day 0, 1, 2 and 3, 10 μL of CCK‐8 solution was added to each well. Following a 2‐h incubation, the absorbance at 450 nm was measured using a microplate reader (Bio‐Tek Instruments Inc., Winooski, VT, USA).

### 2.7 Immunofluorescence (IF)

JVM‐14 and MEC‐1 cells were seeded onto 48‐well plates and incubated at 60°C. Following fixation and permeabilization, cells were incubated with primary anti‐Ki67 antibody (ab243878, 1:100; Abcam) overnight at 4°C. Subsequently, the cells were incubated with a secondary antibody, followed by incubation with DPAI (1:1000 in methanol) for 10 min at 37°C. Fluorescence was observed using an Axio Scope A1 microscope (Zeiss, Germany).

### 2.8 Flow Cytometric Analysis

Cell apoptosis was assessed using Annexin V‐FITC/Propidium Iodide (PI) staining (Sangon) followed by flow cytometry. Briefly, JVM‐14 and MEC‐1 cells were harvested 48 h post‐transfection, resuspended in binding buffer at a concentration of 1 × 10^6^ cells/mL. A 100 μL aliquot of the cell suspension was incubated with 5 μL of Annexin V‐FITC and 5 μL of PI in the dark at room temperature for 15 min. Apoptotic cells were quantified using a FACSCalibur flow cytometer (BD Biosciences, Franklin Lakes, NJ, USA) and analyzed with FACS Diva software. Cells were classified into four quadrants: necrotic cells (Q1), late apoptotic cells (Q2), early apoptotic cells (Q3), and live cells (Q4). The apoptotic rate was calculated as the combined percentage of late apoptotic (Q2) and early apoptotic (Q3) cells.

### 2.9 Western Blotting

Cells were lysed in RIPA buffer (Sigma‐Aldrich) containing a protease inhibitor (ApexBio Technology, Shanghai, China) for total protein extraction. Protein concentrations were determined using the Enhanced BCA Protein assay kit (Yeasen). Equal amounts of protein (30 μg) were separated by SDS‐PAGE and transferred onto PVDF membranes. Following blocking with 5% skimmed milk, the membranes were incubated overnight at 4°C with primary antibodies against Bax (ab182733, 1:2000; Abcam), SLC37A3 (ab155986, 1:3000; Abcam), Bcl‐2 (ab182858, 1:2000; Abcam), TCF12 (ab70746, 1:5000; Abcam), GAPDH (ab181602, 1:10000; Abcam), RAB22A (ab138505, 1:2000; Abcam), and ANKRD13A (1:1000; Invitrogen). The membranes were then incubated with secondary antibodies for 2 h at room temperature and subsequently washed three times with TBST (Sigma‐Aldrich), each wash lasting 10 min. Immunoreactive bands were detected using enhanced chemiluminescence (Yeasen, Shanghai, China) and imaged with a chemiluminescence detection system (Bio‐Rad, Hercules, CA, USA).

### 2.10 Subcellular Fractionation Assay

Cytoplasmic and nuclear fractions were isolated from JVM‐14 and MEC‐1 cells using the NE‐PER Nuclear and Cytoplasmic Extraction Reagents (Heng Fei Biotechnology, Shanghai, China). RT‐qPCR was subsequently performed to identify the expression levels of the cytoplasmic control (GAPDH), nuclear control (U6), and TUSC7.

### 2.11 Luciferase Reporter Assay

The 3′ UTR sequences of miR‐211‐5p, containing either wild‐type (WT) or mutant (MUT) TUSC7 (SLC37A3) binding sites, were synthesized and inserted into pMIR‐REPORT vectors (YouBio, Hunan, China). These constructs were then co‐transfected with miR‐211‐5p mimics or NC mimics into JVM‐14 and MEC‐1 cells using Lipofectamine 2000. After 48 h, relative luciferase activities were measured using a dual‐luciferase reporter assay kit (Yeasen, Shanghai, China) following the manufacturer's instructions.

### 2.12 RNA Pull Down

Biotinylated TUSC7 (TUSC7 probe‐biotin) and a negative control (TUSC7 probe‐no biotin) (Sangon) were separately transfected into JVM‐14 and MEC‐1 cells. After a 10‐min incubation with Dynabeads M‐280 Streptavidin (Invitrogen), RT‐qPCR was employed to quantify and analyze the bound RNAs.

### 2.13 RNA Immunoprecipitation (RIP) Assay

The Magna RIP RNA‐Binding Protein Immunoprecipitation Kit (Huibai Biotechnology, Liaoning, China) was used for RIP experiments. Cells were lysed with RIP lysis buffer, followed by incubation with RIP buffer containing magnetic beads conjugated to human Ago2 antibodies (Invitrogen) at 2°C. IgG was used as a negative control. After 2 h of incubation, RT‐qPCR analysis was performed to assess the abundance of TUSC7 and miR‐211‐5p, with quantification carried out using a NanoDrop spectrophotometer.

### 2.14 Statistical Analysis

Data are presented as mean ± standard deviation. Statistical analysis was performed using GraphPad Prism 8.0. Differences between groups were analyzed by Student's *t*‐test and one‐way analysis of variance. A *p* value of < 0.05 was considered statistically significant.

## 3 Results

### 3.1 Upregulation of TUSC7 Inhibits Cell Proliferation and Promotes Apoptosis

**FIGURE 1:**
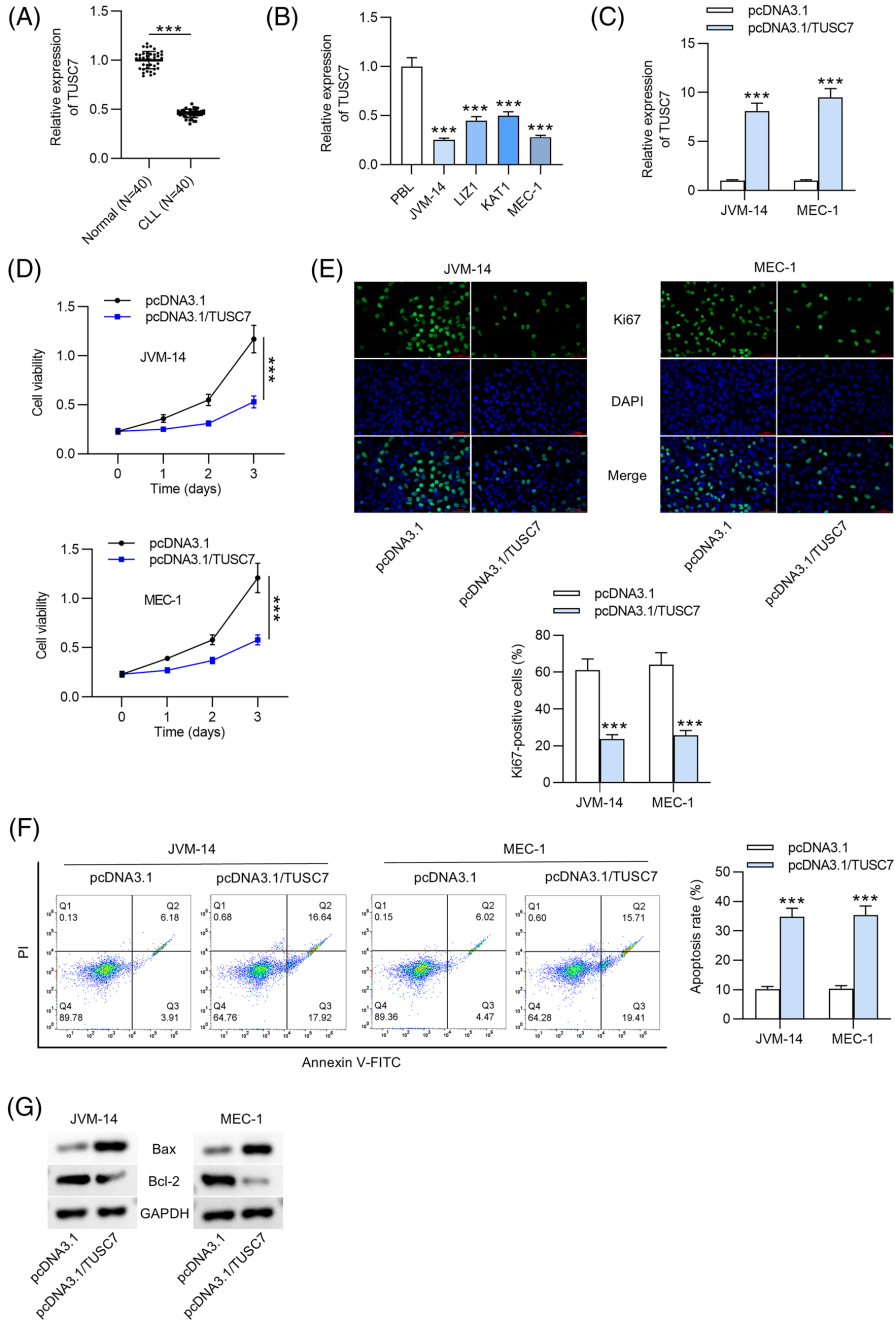
TUSC7 inhibits cell proliferation while exacerbating cell apoptosis. (A) The relative expression of TUSC7 in PBMC samples from 40 treatment‐naïve CLL patients and 40 healthy donors was assessed using RT‐qPCR. (B) TUSC7 expression was confirmed by RT‐qPCR in CLL cells (JVM‐14, MEC‐1, LIZ1, KAT1) and PBLs. (C) Overexpression efficiency of pcDNA3.1/TUSC7 in JVM‐14 and MEC‐1 cells was detected by RT‐qPCR. (D) Cell viability of pcDNA3.1/TUSC7‐transfected cells was determined through CCK‐8 assay. (E) IF assay was implemented to analyze cell proliferation upon upregulated TUSC7. (F) Cell apoptosis upon TUSC7 overexpression was detected via flow cytometric analysis. (G) Protein levels of Bax and Bcl‐2 were measured by western blotting. ***p < 0.001.

Previous studies have demonstrated that lncRNA TUSC7 exerts inhibitory effects in various cancers [18, 20]. In this study, we investigated the biological functions and regulatory mechanisms of TUSC7 in CLL progression. First, we compared the expression levels of TUSC7 between CLL patients and healthy controls. Quantitative reverse transcription PCR (RT‐qPCR) revealed a significant downregulation of TUSC7 in CLL patients (Figure 1A). As shown in Table 1, the downregulation of TUSC7 correlated positively with several adverse clinical parameters, including male gender, positive CD38 status, elevated CLL‐IPI scores, the presence of B symptoms, low platelet count, low hemoglobin levels, and increased LDH activity. These findings suggest that reduced TUSC7 expression may serve as a prognostic marker for poor outcomes in CLL patients. Additionally, TUSC7 downregulation was confirmed in CLL cell lines (JVM‐14, MEC‐1, LIZ1, and KAT1) relative to PBLs (Figure 1B). Then, we effectively upregulated TUSC7 expression using the pcDNA3.1/TUSC7 vector, with pcDNA3.1 serving as a scramble control. The overexpression efficiency was confirmed through RT‐qPCR analysis (Figure 1C). In terms of biological functions, CCK‐8 assays demonstrated a significant reduction in the viability of JVM‐14 and MEC‐1 cells following TUSC7 overexpression (Figure 1D). IF staining revealed a marked decrease in the number of Ki67‐positive JVM‐14 and MEC‐1 cells in the TUSC7 upregulation group compared to the negative control group (Figure 1E). Additionally, flow cytometry analysis showed that TUSC7 upregulation significantly enhanced apoptosis in JVM‐14 and MEC‐1 cells (Figure 1F). These findings were corroborated by Western blot analysis, which indicated a notable increase in Bax protein levels and a substantial decrease in Bcl‐2 protein levels in TUSC7‐overexpressing CLL cells compared to control CLL cells (Figure 1G). Collectively, these results demonstrate that TUSC7 expression is significantly downregulated in CLL patients and CLL cells, and that its upregulation inhibits cell proliferation while promoting apoptosis in CLL cells.

### 3.2 MiR‐211‐5p, Identified as an Upregulated miRNA in CLL, Is Downstream of TUSC7

**FIGURE 2:**
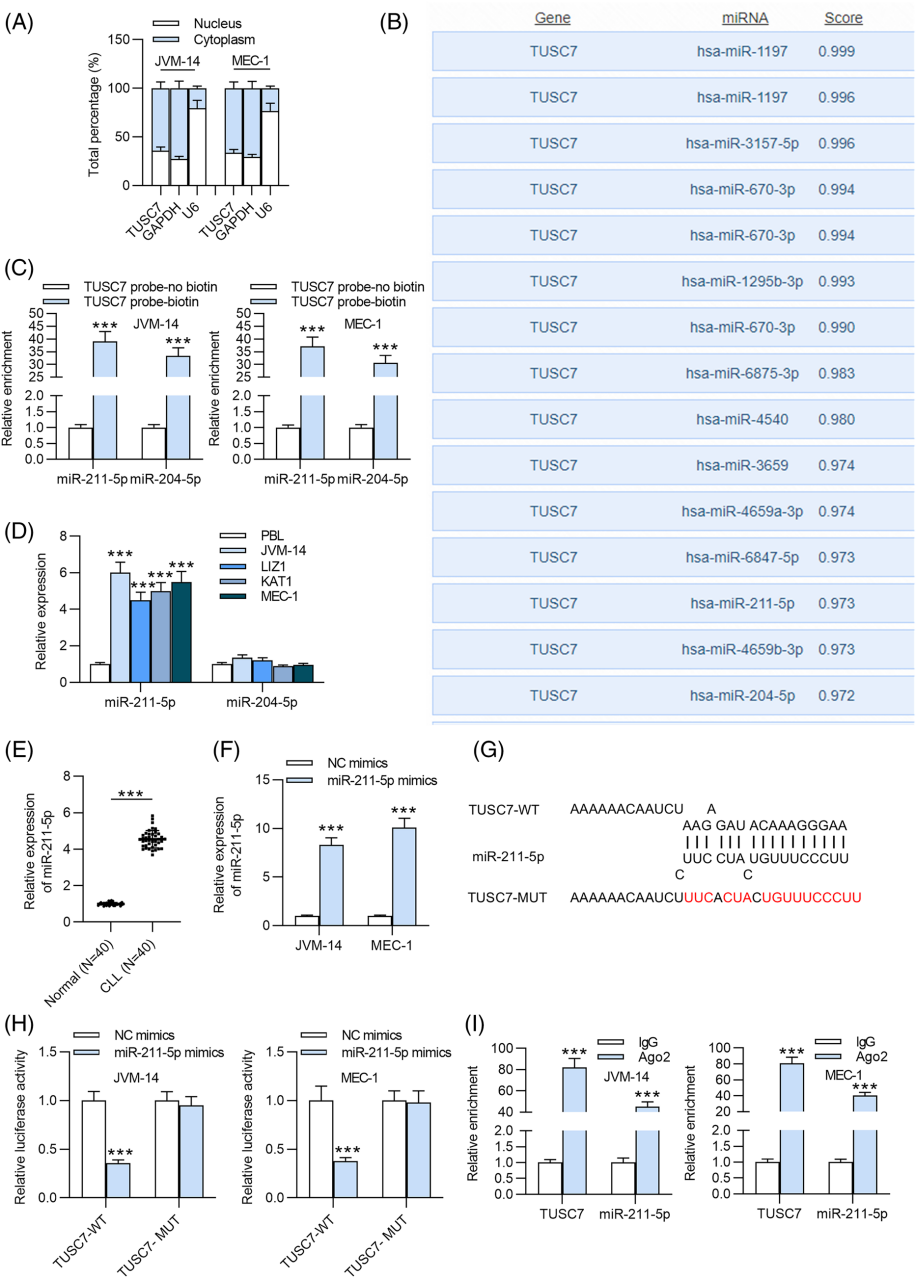
MiR‐211‐5p, identified as an upregulated miRNA in CLL, is a downstream target of TUSC7. (A) Subcellular fractionation assay was performed to determine the localization of TUSC7. (B) Prediction of potential target of TUSC7 was conducted by online tool DIANA database. (C) Pull down assay was applied for filtering the qualified target of TUSC7. (D) Relative expression of two candidate miRNAs were detected with RT‐qPCR in CLL cells (JVM‐14, MEC‐1, LIZ1, KAT1) and PBLs to select the qualified target for the subsequent experiments. (E) RT‐qPCR was conducted to detect miR‐211‐5p level in PBMC samples from 40 treatment‐naïve CLL patients and 40 normal donors. (F) The overexpression efficiency of miR‐211‐5p mimics was confirmed by RT‐qPCR. (G) Prediction of target sequence of miR‐211‐5p on the 3′‐UTR of TUSC7 was carried out via online tool DIANA database. (H) Luciferase reporter assay was used to confirm the interaction between TUSC7 and miR‐211‐5p in CLL cells. (I) RIP assay was performed to assure the ability of TUSC7 to serve as miR‐211‐5p sponge. ***p < 0.001.

We then investigated the regulatory mechanism of TUSC7 in CLL progression. As previously reported, cytoplasmic lncRNAs harbor sequences that are complementary to miRNAs, enabling them to directly or indirectly regulate miRNA expression through the ceRNA network [27]. Accordingly, we assessed the subcellular localization of TUSC7. Subcellular fractionation assays revealed that TUSC7 was predominantly located in the cytoplasm of JVM‐14 and MEC‐1 cells, suggesting its potential role as a ceRNA (Figure 2A). Based on these findings, we hypothesized that TUSC7 acts as a tumor suppressor by interacting with miRNAs in CLL cells. To predict the downstream targets of TUSC7, we utilized the DIANA database, which identified the top 15 miRNAs with the highest binding scores as potential candidates (Figure 2B). To validate the interaction between these candidate miRNAs and TUSC7, we conducted RNA pull‐down assays, which revealed that only miR‐211‐5p and miR‐204‐5p exhibited abundant binding with TUSC7 (Figure 2C). Subsequently, RT‐qPCR was employed to quantify the expression levels of miR‐211‐5p and miR‐204‐5p in PBLs and CLL cells. The results showed that miR‐211‐5p was significantly upregulated in CLL cells compared to PBLs, whereas no significant changes were observed in miR‐204‐5p expression across the groups (Figure 2D). Thus, miR‐211‐5p was selected for further investigation. The results of RT‐qPCR revealed that miR‐211‐5p was significantly upregulated in patients with CLL compared to healthy individuals (Figure 2E). To further investigate, we successfully overexpressed miR‐211‐5p in JVM‐14 and MEC‐1 cells using miR‐211‐5p mimics, and the overexpression efficiency was confirmed by RT‐qPCR (Figure 2F). We then utilized the DIANA database to predict potential binding sites between TUSC7 and miR‐211‐5p (Figure 2G). Luciferase reporter assays revealed that the upregulation of miR‐211‐5p significantly inhibited the luciferase activity of the TUSC7‐WT construct compared to the control, whereas no significant change was observed for the TUSC7‐MUT construct (Figure 2H). Additionally, RIP assays demonstrated that both TUSC7 and miR‐211‐5p were enriched in Ago2‐conjugated beads compared to normal IgG controls (Figure 2I). Collectively, these results indicate that TUSC7 directly interacts with miR‐211‐5p.

### 3.3 MiR‐211‐5p Targets SLC37A3 3’UTR

**FIGURE 3:**
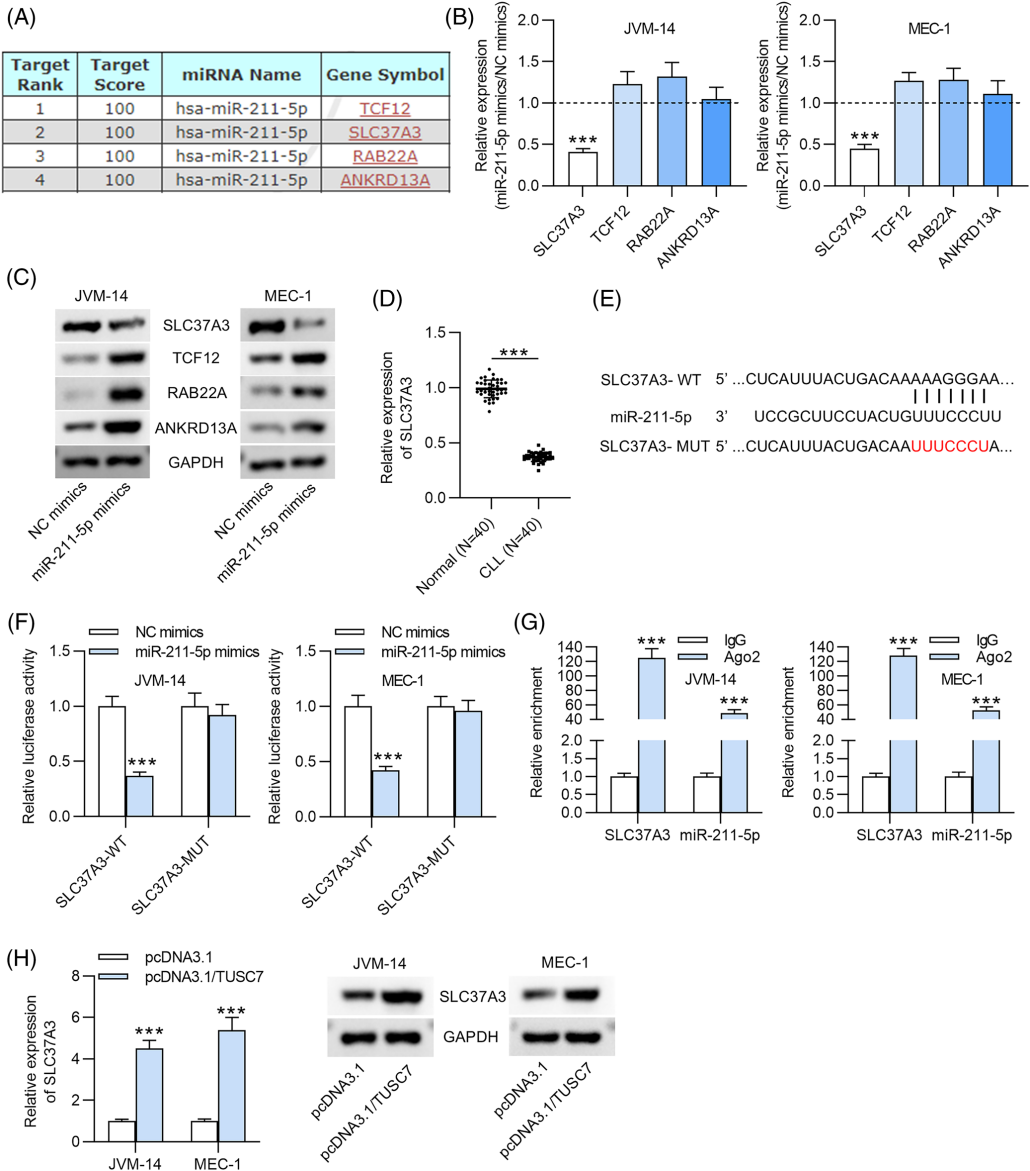
MiR‐211‐5p targets SLC37A3. (A) Online tool miRDB database was utilized for prediction of potential target of miR‐211‐5p. (B) RT‐qPCR was used to assess the mRNA levels of four candidate targets following miR‐211‐5p overexpression. (C) Western blot analysis was performed to measure protein levels. (D) RT‐qPCR was used to evaluate the relative expression of SLC37A3 in PBMC samples from 40 treatment‐naïve CLL patients and 40 healthy donors. (E) The target sequence of miR‐211‐5p on the 3′‐UTR of SLC37A3 was predicted by online tool TargetScan database. (F) The interaction between SLC37A3 and miR‐211‐5p in CLL cells was verified with luciferase reporter assay. (G) Ability of TUSC7 to serve as miR‐211‐5p sponge was confirmed by RIP assay. (H) RT‐qPCR and western blot analysis were respectively carried out to measure relative expression and protein levels of SLC37A3 in each group. ***p < 0.001.

To identify potential target genes of miR‐211‐5p, predictions were made using the miRDB database. SLC37A3, TCF12, RAB22A and ANKRD13A were initially identified as candidate targets of miR‐211‐5p (Figure 3A). RT‐qPCR and western blot analysis revealed that only the mRNA and protein levels of SLC37A3 were significantly reduced in response to miR‐211‐5p overexpression (Figure 3B,C). Consequently, SLC37A3 was selected for further investigation as a potential target of miR‐211‐5p. RT‐qPCR analysis demonstrated that SLC37A3 expression was notably lower in CLL patients compared to healthy individuals (Figure 3D). We further predicted the putative binding sites of miR‐211‐5p in the 3′UTR of SLC37A3 using the TargetScan database (Figure 3E). To confirm the interaction between miR‐211‐5p and SLC37A3, luciferase reporter assays were performed. The results showed a significant reduction in luciferase activity in cells transfected with the SLC37A3‐WT construct upon miR‐211‐5p mimic transfection, whereas no change in luciferase activity was observed in the SLC37A3‐MUT group (Figure 3F). Additionally, RIP assays demonstrated significant enrichment of both SLC37A3 and miR‐211‐5p in the Ago2 complex (Figure 3G). Moreover, overexpression of TUSC7 led to a marked increase in both the mRNA and protein levels of SLC37A3 (Figure 3H). These results collectively suggest that miR‐211‐5p directly targets the 3′UTR of SLC37A3.

### 3.4 TUSC7 Inhibits Cell Proliferation via the miR‐211‐5p/SLC37A3 Axis

**FIGURE 4:**
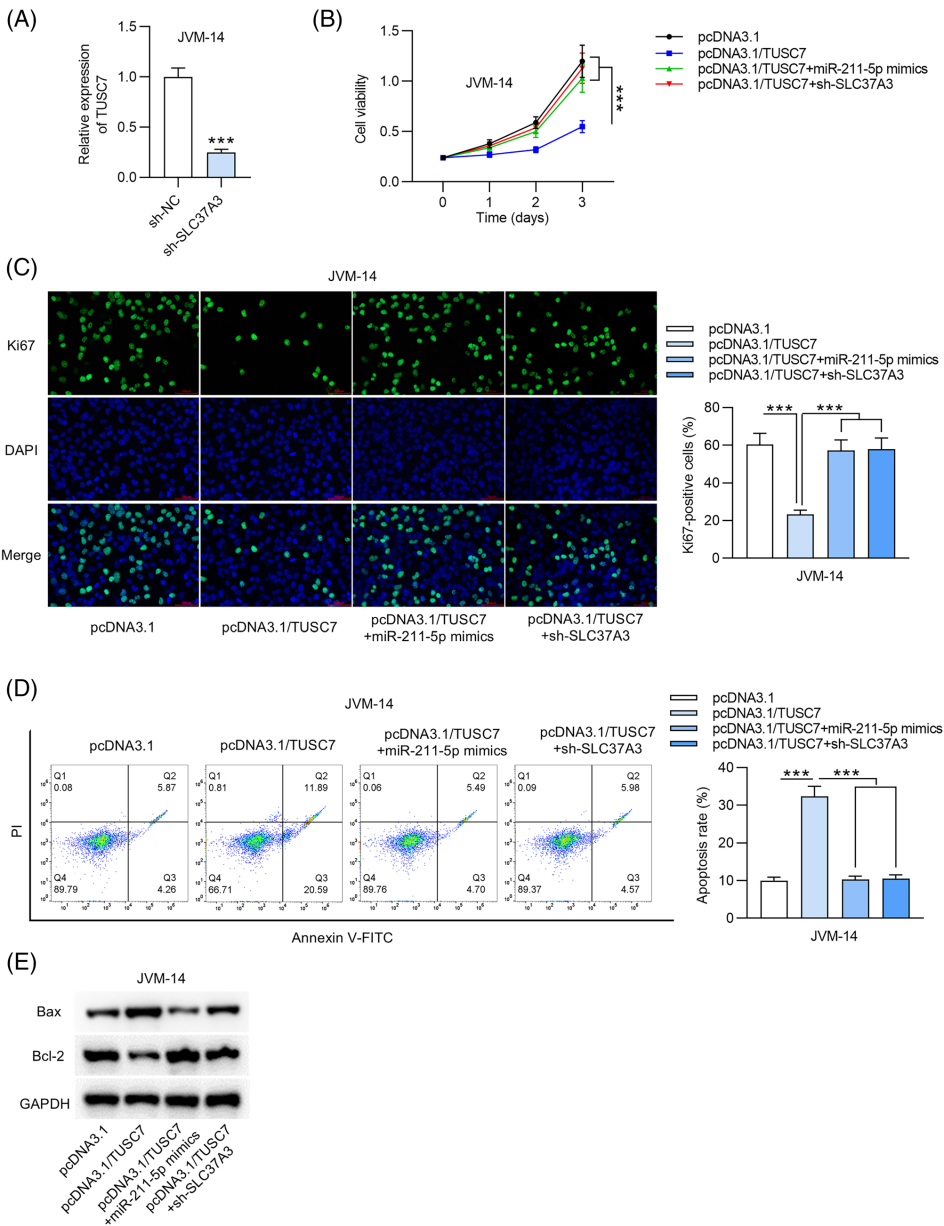
TUSC7 inhibits cell proliferation through the miR‐211‐5p/SLC37A3 axis. (A) The knockdown efficiency of sh‐SLC37A3 in JVM‐14 cells was assessed by RT‐qPCR. (B) Viability of JVM‐14 cells in each group was measured by CCK‐8 assay. (C) IF assay was performed to assure cell proliferation. (D) Cell apoptosis was verified by flow cytometric analysis. (E) Western blotting was used to measure protein levels of apoptosis markers (Bax and Bcl‐2) in each transfected group. ***p < 0.001.

Finally, we assessed whether TUSC7 inhibited proliferation and promoted apoptosis in CLL cells through the miR‐337‐3p/SLC37A3 axis. Initially, we effectively knocked down SLC37A3 in JVM‐14 cells, with knockdown efficiency confirmed by RT‐qPCR (Figure 4A). CCK‐8 assays revealed that either SLC37A3 knockdown or upregulation of miR‐211‐5p counteracted the suppressive effect of TUSC7 upregulation on cell viability (Figure 4B). Moreover, downregulation of SLC37A3 or upregulation of miR‐211‐5p rescued the TUSC7‐mediated decrease in the number of Ki67‐positive CLL cells (Figure 4C). Flow cytometric analysis further showed that downregulation of SLC37A3 or upregulation of miR‐211‐5p reversed the TUSC7‐induced promotion of cell apoptosis (Figure 4D). Simultaneously, we found that the effects of pcDNA3.1/TUSC7 on Bax and Bcl‐2 levels were abolished following transfection with sh‐SLC37A3 or miR‐211‐5p mimics (Figure 4E). These findings show that TUSC7 inhibits CLL progression via the miR‐211‐5p/SLC37A3 axis.

## 4 Discussion

It has become increasingly evident that lncRNAs, as emerging ncRNAs, play critical roles in modulating cell proliferation and apoptosis [28, 29]. Notably, TUSC7 has been identified as significantly downregulated in various cancers, demonstrating tumor suppressive effects, including in osteosarcoma [18, 19], endometrial carcinoma [20], colorectal cancer [21, 22], and pancreatic carcinoma [23]. Our findings further support this notion, as TUSC7 was markedly downregulated in CLL, with its downregulation correlating with poor prognosis in CLL patients. Moreover, TUSC7 functions as a tumor suppressor by inhibiting cell proliferation and promoting apoptosis in CLL.

According to cytoplasmic localization of TUSC7, we explored its post‐transcriptional regulation of gene expression. As previously described, ceRNAs are transcripts that interact at the post‐transcription level by competing for shared miRNAs [30]. Previous studies have suggested the ceRNA mechanism of TUSC7 in various cancers. For instance, in triple‐negative breast cancer, TUSC7 inhibits metastasis by binding to miR‐1224‐3p [31]. Additionally, TUSC7 suppresses the proliferation and migration of osteosarcoma cells through its interaction with miR‐375 [32]. Moreover, TUSC7 attenuates pancreatic cancer progression by regulating miR‐371a‐5p [33]. These findings suggest a potential ceRNA regulatory mechanism for TUSC7. Through bioinformatics analysis, we identified miR‐211‐5p as a downstream target of TUSC7 in CLL. Notably, miR‐211‐5p has been implicated in the progression of lung adenocarcinoma [34] and non‐small cell lung cancer [35]. Conversely, miR‐211‐5p has been shown to mitigate the malignant phenotypes of thyroid cancer [36], prostate cancer [37], and hepatocellular carcinoma cells [38]. In our study, we found that miR‐211‐5p was significantly upregulated in CLL, and its overexpression reversed the inhibitory effects of TUSC7 upregulation on CLL progression.

To validate the ceRNA model, we utilized bioinformatics to identify potential targets of miR‐211‐5p. SLC37A3, an endoplasmic reticulum (ER)‐associated sugar‐phosphate/phosphate exchanger [39], has not been previously reported in cancers. In this study, we found that SLC37A3 expression was significantly reduced in patients with CLL. Furthermore, knockdown of SLC37A3 was shown to reverse the inhibitory effects of TUSC7 upregulation on cell proliferation, while promoting cell apoptosis.

Taken together, our results demonstrate that both TUSC7 and SLC37A3 expression levels are downregulated in CLL, while miR‐211‐5p is upregulated. Furthermore, we show that TUSC7 acts as a sponge for miR‐211‐5p, thereby regulating the expression of SLC37A3 and influencing cell proliferation and apoptosis. Despite these insights, there are several limitations to this study. First, in vivo experiments were not performed. Second, further investigation into the downstream signaling pathways is required. Nevertheless, this study introduces a novel biomarker for CLL and holds significant clinical potential for improving treatment strategies.

## Ethics Statement

The Ethics Review Board of The Second Hospital of Nanjing, Affiliated to Nanjing University of Chinese Medicine approved the study.

## Conflicts of Interest

The authors declare no conflicts of interest.

## Data Availability

The data that support the findings of this study are available from the corresponding author upon reasonable request.
